# Exposure to the gut microbiota drives distinct methylome and transcriptome changes in intestinal epithelial cells during postnatal development

**DOI:** 10.1186/s13073-018-0534-5

**Published:** 2018-04-13

**Authors:** Wei-Hung Pan, Felix Sommer, Maren Falk-Paulsen, Thomas Ulas, Lena Best, Antonella Fazio, Priyadarshini Kachroo, Anne Luzius, Marlene Jentzsch, Ateequr Rehman, Fabian Müller, Thomas Lengauer, Jörn Walter, Sven Künzel, John F. Baines, Stefan Schreiber, Andre Franke, Joachim L. Schultze, Fredrik Bäckhed, Philip Rosenstiel

**Affiliations:** 1 Institute for Clinical Molecular Biology, University of Kiel, Rosalind-Franklin-Straße 12, 24105 Kiel, Germany; 2 The Wallenberg Laboratory, Department of Molecular and Clinical Medicine, University of Gothenburg, 41345 Gothenburg, Sweden; 3 Genomics and Immunoregulation, LIMES-Institute, University of Bonn, 53115 Bonn, Germany; 4 Max Planck Institute for Informatics, 66123 Saarbrücken, Germany; 5 Graduate School of Computer Science, Saarland University, 66123 Saarbrücken, Germany; 6 Department of Genetics, University of Saarland, 66123 Saarbrücken, Germany; 7 Institute for Experimental Medicine, Christian Albrechts University of Kiel, Kiel, Germany; 8 Max Planck Institute for Evolutionary Biology, Evolutionary Genomics, August-Thienemann-Str. 2, 24306 Plön, Germany; 9 Department of Internal Medicine I, University Hospital Schleswig Holstein, 24105 Kiel, Germany; 10 Platform for Single Cell Genomics and Epigenomics (PRECISE), German Center for Neurodegenerative Diseases and the University of Bonn, Bonn, Germany; 11 Novo Nordisk Foundation Center for Basic Metabolic Research, Section for Metabolic Receptology and Enteroendocrinology, Faculty of Health Sciences, University of Copenhagen, 2200 Copenhagen, Denmark

**Keywords:** Microbiota, Intestinal epithelial cell, Epigenetics, Methylation, Transcriptomics

## Abstract

**Background:**

The interplay of epigenetic processes and the intestinal microbiota may play an important role in intestinal development and homeostasis. Previous studies have established that the microbiota regulates a large proportion of the intestinal epithelial transcriptome in the adult host, but microbial effects on DNA methylation and gene expression during early postnatal development are still poorly understood. Here, we sought to investigate the microbial effects on DNA methylation and the transcriptome of intestinal epithelial cells (IECs) during postnatal development.

**Methods:**

We collected IECs from the small intestine of each of five 1-, 4- and 12 to 16-week-old mice representing the infant, juvenile, and adult states, raised either in the presence or absence of a microbiota. The DNA methylation profile was determined using reduced representation bisulfite sequencing (RRBS) and the epithelial transcriptome by RNA sequencing using paired samples from each individual mouse to analyze the link between microbiota, gene expression, and DNA methylation.

**Results:**

We found that microbiota-dependent and -independent processes act together to shape the postnatal development of the transcriptome and DNA methylation signatures of IECs. The bacterial effect on the transcriptome increased over time, whereas most microbiota-dependent DNA methylation differences were detected already early after birth. Microbiota-responsive transcripts could be attributed to stage-specific cellular programs during postnatal development and regulated gene sets involved primarily immune pathways and metabolic processes. Integrated analysis of the methylome and transcriptome data identified 126 genomic loci at which coupled differential DNA methylation and RNA transcription were associated with the presence of intestinal microbiota. We validated a subset of differentially expressed and methylated genes in an independent mouse cohort, indicating the existence of microbiota-dependent “functional” methylation sites which may impact on long-term gene expression signatures in IECs.

**Conclusions:**

Our study represents the first genome-wide analysis of microbiota-mediated effects on maturation of DNA methylation signatures and the transcriptional program of IECs after birth. It indicates that the gut microbiota dynamically modulates large portions of the epithelial transcriptome during postnatal development, but targets only a subset of microbially responsive genes through their DNA methylation status.

**Electronic supplementary material:**

The online version of this article (10.1186/s13073-018-0534-5) contains supplementary material, which is available to authorized users.

## Background

A tremendously complex and dynamic union of microorganisms inhabits the mammalian gastrointestinal tract and contributes to several aspects of host physiology, including metabolism, maturation of the immune system, cellular homeostasis, and behavior [1–3]. However, the commensal microbial communities within the host also represent a danger due to their potential for infection and overgrowth. Thus, mechanisms are in place to assure a healthy beneficial coexistence. Intestinal epithelial cells (IECs) take a central role as they line the gastrointestinal mucosa and build a physicochemical and immunological barrier to restrain the microbiota and prevent invasion [4, 5]. Interactions between the microbiota and the host, especially IECs, have therefore been studied intensively in the past decade [6–11]. Previous studies have shown that under normal homeostatic conditions the gut microbiota regulates the expression of about 10% of host genes [6]. Several mechanisms have been implicated in how the gut microbiota can drive these global changes in the host transcriptome. Transcriptional regulators such as NFκB (nuclear factor kappa-light-chain-enhancer of activated B cells) or CEBPB (CCAAT/enhancer-binding protein beta) may be engaged by the microbiota to modulate the expression of specific target genes [6, 12]. Additionally, the microbiota have the potential to modulate host epigenetic mechanisms and thereby regulate transcription more globally [13–18]. The microbially produced short-chain fatty acids (SCFAs) butyrate and propionate are potent inhibitors of histone deacetylase (HDAC) enzymes [14] and therefore may promote heterochromatin formation and increase transcriptional activity. However, global changes in the accessible chromatin landscape by the gut microbiota were not detected in a previous study [12]. Additionally, the intestinal microbiota may modulate DNA methylation, since microbially produced folate is an essential methyl donor during DNA methylation [16].

DNA methyltransferases (DNMT) catalyze the transfer of the methylation group from methionine to cytosine if it is followed by a guanine (CpG). DNMT1 maintains the methylation pattern during DNA replication [19] whereas DNMT3a and DNMT3b perform de novo methylation [20]. DNA methylation occurs predominantly at a series of two or more CpGs [21–23]. DNA methylation is thought to inhibit gene transcription, but recent data indicate that the functional consequences may be more complex [24] and depend at least partially on the location of the methylated site. If 5-methylcytosine is situated in close vicinity to a transcription start site, transcription of the downstream gene is mainly blocked [25]. In contrast, methylation of CpGs in the gene body may rather influence transcript elongation or splicing [26]. DNA methylation plays a key role during development and cellular differentiation function [25, 27]. DNA methylation is mostly erased during zygote formation and reprogrammed during development [28]. Yu and colleagues have shown that during postnatal development both the epithelial transcriptome and the DNA methylation landscape undergo fundamental reshaping [29]. The early neonatal period is a critical phase not only for the development of the intestinal tract but also for the establishment of the microbiota and proper maturation of the immune system [30, 31]. A series of reports established the presence of a window of opportunity based on observations that lack of exposure to environmental microbes during early development may lead to immunological defects and autoimmune diseases later in life [32–37]. Notably, colonization at a later stage fails to normalize these immunological defects. This persistence of microbiota-dependent regulatory signatures points to microbial imprinting through epigenetic mechanisms (possibly DNA methylation) that are long lasting once they are established [2, 17]. However, whether microbial colonization early in life alters the DNA methylation pattern and alongside the epithelial transcriptome during postnatal development and maturation of the gut epithelium remains largely unknown. To address this issue, we collected IECs from the small intestine of 1-, 4- and 12 to 16-week-old mice, which were raised in either the presence or absence of a microbiota to represent the infant, juvenile, and adult states of the epithelium and the intestinal flora. We then measured the methylation variable positions using reduced representation bisulfite sequencing (RRBS) and analyzed the epithelial transcriptome by RNA sequencing (RNA-Seq) to investigate the association between gene expression, alternative splicing, and differential DNA methylation in IECs during postnatal ontogeny.

## Methods

### Mice

C57Bl6/N female littermate mice were maintained under standard specific pathogen-free or germ-free (GF) conditions in the laboratory for experimental biomedicine at University of Gothenburg as described previously [38]. Mice were kept under a 12-h light cycle and fed autoclaved chow diet *ad libitum* (Labdiet, St Louis, MO, USA). Mice were sacrificed at three different stages: 1, 4 and between 12 to 16 weeks of age with *n* = 5 animals for each of the groups. Mice were killed by cervical dislocation and the small intestine removed for isolation of IECs. All animal protocols were approved by the Gothenburg Animal Ethics Committee.

### Isolation of IECs

IECs were isolated from small intestinal tissue using the Lamina Propria Dissociation Kit (Miltenyi BioTech, Bergisch Gladbach, Germany) according to the manufacturer’s protocol. In brief, intestinal epithelial cells were isolated by disruption of the structural integrity of the epithelium using ethylenediaminetetraacetic acid (EDTA) and dithiothreitol (DTT). Purity of individual IEC fractions was analyzed by flow cytometry on a FACS Calibur flow cytometer (B&D, Heidelberg, Germany) with Cellquest analysis software from Becton Dickinson. We used the Anti-EpCam-PE (clone G8.8, Biolegend, San Diego, USA) antibody for analysis of IEC purity.

### Transcriptional profiling by RNA sequencing

RNA was isolated from purified small intestinal IECs using the TRIZOL method. Briefly, 1 ml TRIzol was added to 50–75 mg pestle-homogenized tissue followed by vortexing, a 5-min incubation at room temperature, and addition of 200 μl chloroform. After mixing, further incubation at room temperature for 2–3 min and centrifugation (12.000 g) at 4 °C for 5 min, the clear supernatant was mixed with 500 μl isopropanol followed by incubation at room temperature for 10 min. After further centrifugation (12.000 g) at 4 °C for 10 min, the supernatant was discarded and the pellet washed with 1 ml cold 75% EtOH followed by vortexing and centrifugation (7.500 g, 4 °C, 5 min). The pellet was dried and dissolved in RNase-free water. RNA libraries were prepared using TruSeq v4 Kit (Illumina) according to the manufacturer’s instructions. All samples were sequenced using an Illumina HiSeq 2000 sequencer (Illumina, San Diego,CA) with an average of 23 million paired-end reads (2 × 125 bp) at IKMB NGS core facilities. We used TopHat 2 [39] and Bowtie 2 [40] to align reads. Reads were mapped to the mouse genome (MGI assembly version 10) using TopHat 2. Average alignment rate for RNA-seq was 83.3% (73.3–89.9%, median = 85.7%) and the expression count was normalized by library size. Gene expression values of the transcripts were computed by HTSeq [41]. DEseq2 [42] was used to determine differentially expressed genes. Genes were considered as significant differentially expressed if the adjusted *p* value (Benjamini–Hochberg (BH) multiple test correction method) was less than 0.05. Gene expression differences were visualized using MA plot [43], a modification of a Bland–Altman plot for visual representation of genome-wide functional genomic data. M represents the log fold change for gene expression (y-axis) and A represents the mean normalized counts (x-axis). We’ve set the ceiling/floor to 2 on log fold change (y-axis) to achieve an optimal visualization. PCA was performed using plotpca in the R package DEseq2 and Euclidian distance was measured. Transcription factor binding site analysis was carried out using the Innate DB database [44] with implementation of the hypergeometric algorithm and the BH multiple test correction method (BH-corrected *p* value < 0.05). Only expressed transcription factors were considered for the analysis (raw read count > 3). Gene Ontology (GO) analysis was performed using the GOrilla (gene ontology enrichment analysis and visualization) tool [45]. GO terms with false discovery rate (FDR) < 0.05 were considered significantly altered. All RNA-Seq data have been uploaded to the Gene Expression Omnibus (GEO) with accession number GEO:GSE94402.

### Co-expression network analysis

For the establishment of a gene co-expression network, we built the union of differentially expressed genes comparing always the conventionally raised specific pathogen-free (CONV-R) and GF conditions at the same time point. Expression values of these genes over all 30 samples were used for the co-expression analysis using BioLayout Express 3D [46]. Applying a correlation cutoff of 0.8 resulted in a co-expression network with 970 nodes (genes) and 34,437 edges. The calculated gene–gene pairs and their Spearman correlation coefficients were imported into Cytoscape using organic layout for visualization. Subsequently, we mapped condition fold changes (based on the comparison of each condition with the mean of all conditions) individually for each condition onto the network, to identify condition-specific topological differences between the conditions in the co-expression network. Gene groups were assigned based on the temporal and microbiota-dependent expression changes with the following specific criteria: group 1, expressed high (Z-score > + 1 in condition gene expression value normalized by the mean condition value) in W1, low (condition Z-score < − 1) in W4 + W12/16, independent of GF/CONV-R; group 2, expressed high in W12 CONV-R but low in W12 GF, normal (condition Z-score − 1 to + 1) in W1 and W4 CONV-R, low in W4 GF; group 3, expressed high in W12 CONV-R but low in W12 GF, low in W1 + W4; group 4, expressed high in W12 CONV-R but low in W12 GF, low in W1 + W4; group 5, expressed high in W12 GF but low in W12 CONV-R, high in W4 GF, low in W1 GF, W1 CONV-R, and W4 CONV-R; group 6, expressed high in W12 GF but low in W12 CONV-R, high in W4 GF, low in W4 CONV-R, normal in W1 GF + CONV-R.

### Transcript splicing analysis

Based on the updated genome annotation and our RNA-Seq data, we compared the alternative splicing events of each gene between CONV-R and GF in three stages. We used rMATS [47], which detects alternative splicing events such as skipped exons, alternative 5′ splice sites, alternative 3′ splice sites, mutually exclusive exons, and retained intron events. The events were identified as significantly different by choosing inclusion levels of |ΔPSI| ≥ 5% between CONV-R and GF at FDR q < 0.05.

### Reduced representation bisulfite sequencing

DNA was isolated from purified IECs using a DNeasy Blood & Tissue Kit (Qiagen) according to the manufacturer’s instructions. DNA libraries were sequenced at IKMB NGS core facilities using Illumina HiSeq 2500 sequencer (Illumina, San Diego, CA, USA) with an average of 127,000,000 single-end 50-bp reads. After removing adaptor sequences and low-quality tails, reads were mapped to the mouse genome (MGI version 10) using Bismark [48]. All CpG sites covered by less than five reads were removed along with SNPs specific to the C57BL/6 N strain (http://www.sanger.ac.uk/science/data/mouse-genomes-project). We used MethylKit [49] for gene category and CGI annotation and downloaded the gene information from Refseq. The average mapping efficiency of reduced representation bisulfite sequencing (RRBS) was 71.37% (63–78.28%, median = 70.73%). We used Dispersion shrinkage for sequencing data [50, 51] to identify differentially methylated loci based on a beta-binomial regression model with “arcsine” link function. Parameter estimation was based on transformed data with a generalized least square approach without relying on an iterative algorithm. One CONV-R W1 sample was excluded from the DNA methylation analysis due to failure of the bisulfite conversion. All RRBS data have been uploaded to GEO with accession number GEO:GSE94402.

### Integrated analysis screening for differentially methylated and expressed genes

For integrated analysis of gene expression and DNA methylation, we applied a hierarchical testing approach [52] to detect DNA methylation sites around the differentially expressed gene. To that end, we identified all CpG sites 5 kb up- and downstream of the transcription start site of the microbially regulated genes. Second, we combined the neighborhood methylation positions to methylation regions (maximum distance 200 bp). Those regions, which contained less than 20% CpGs (BH-corrected *p* value < 0.05), were excluded and all retained regions were considered as differentially methylated regions. FDR correction was performed on all CpGs of the retained regions (BH-corrected *p* value < 0.05). The R code used for the integrated analysis is included in Additional file 1. The circular visualization plot was constructed using the R package *circlize* [53].

### Functional network analysis for differentially methylated and expressed genes

To screen for functional networks among the differentially methylated and expressed genes (CONV-R versus GF) we employed the Functional Networks of Tissues in Mouse [54] prediction tool for mouse tissue-specific protein interactions, which integrates genomic data and prior knowledge of gene function. We used the small intestine tissue database and only kept edges with relationship confidence greater than 0.6.

### Validation of identified microbiota-dependent genes and differentially methylated positions

To validate our findings in an independent set of animals, we isolated DNA and RNA from small intestinal epithelial scrapings of 4- and 12-week-old GF and CONV-R C57Bl6 mice (*n* = 10 per group) from the gnotobiotic animal facility of the Max Planck Institute for Evolutionary Biology in Plön, Germany. Among all of the genes with differential expression and methylation, we selected 3 out of 34 for W4 (Bcl3, Nfix, Cacnali) and 5 out of 79 for W12/16 (Rcbtb2, Mmp14, Itga5, Cd74, Pik3cd) based on the following criteria for the validation experiment: BH-corrected *p* value among the most significant; fold change among the most differential; validated qPCR primers available in either published studies or public databases.

For qPCR analysis, 1 μg of total RNA was reverse-transcribed to cDNA according to the manufacturer’s instructions (MultiScribe Reverse Transcriptase; Applied Biosystems). qPCR was carried out using SYBR Select Master Mix (Applied Biosystems) according to the manufacturer’s instructions. Primer sequences are given in Additional file 2. Reactions were carried out on the 7900HT Fast Real Time PCR System (Applied Biosystems). Expression levels were normalized to β-actin.

Region and base-specific methylation information was obtained via Bisulfite Amplicon Sequencing. This protocol involved bisulfite conversion of sample DNA (EpiTect Bisulfite Kit, QIAGEN) followed by PCR-amplification of target differentially methylated position (DMP)-containing regions (EpiMark Hot Start Taq, NEB). Primer pairs were designed using “MethPrimer” [55] and target specificity was evaluated using “BiSearch” [56]. PCR amplicons were normalized using SequalPrep plates (ThermoFisher), pooled sample-wise, and subjected to NGS library preparation (Nextera XT, Illumina) according to the manufacturer’s instructions. Finally, the library pool was sequenced on a MiSeq platform (Illumina) with 150-bp, paired-end reads. Raw reads were trimmed for adapter and transposon sequences and only bases with a quality value below 30 were kept using Cutadapt 1.10. Reads were then mapped by Bismark 0.15.0 [48] with Bowtie 2.2.5 [40] to the mouse reference genome (mm10). Methylation ratios were extracted using Bismark and analyzed using R with the package bsseq [57].

## Results

### The gut microbiota and chronological age determine the epithelial transcriptome during postnatal development

**Fig. 1:**
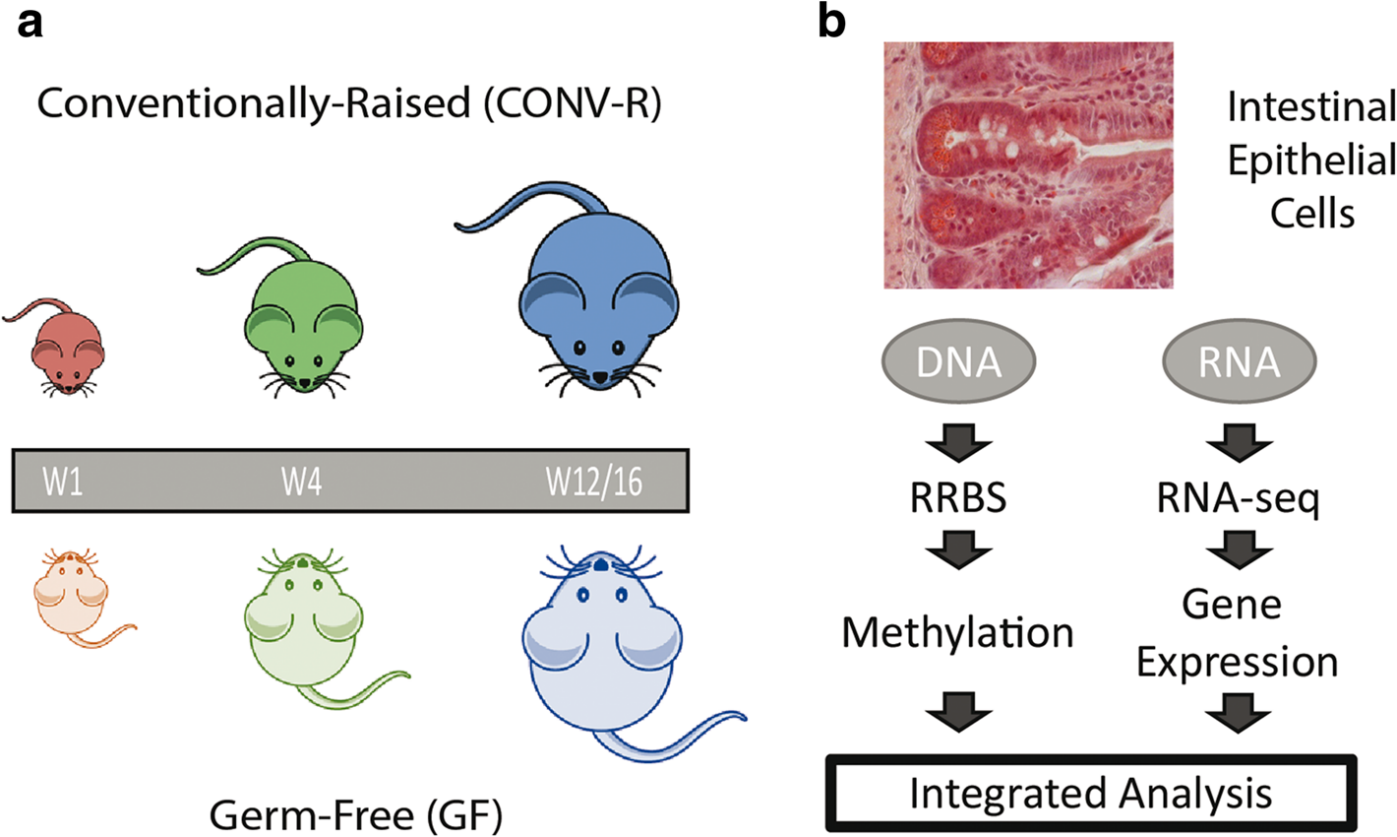
Experimental study design. **a** Mice that were raised conventionally (*CONV-R*) or germ-free (*GF*) were sacrificed at three developmental stages: 1 week, 4 weeks, and between 12 and 16 weeks of age. **b** Intestinal epithelial cells (IECs) from the distal small intestine were collected. DNA and RNA were isolated and gene expression and DNA methylation analyzed by RNA-seq and RRBS, respectively

To investigate potential effects of the gut microbiota and postnatal development on dynamic host epigenetic signatures and changes in the transcriptional profiles of the epithelial cells, we isolated DNA and RNA from IECs of conventionally raised and germ-free C57BL6 female mice (*n* = 5 per group) at three different stages during postnatal development—week 1, week 4, and week 12/16 (W1, W4, W12/16)—representative of the infant, juvenile, and adult states (Fig. 1a), respectively. RNA and DNA were isolated and subjected to RNA-Seq and RRBS to assess global mRNA expression and DNA methylation profiles, respectively (Fig. 1b). After quality control and data pre-processing, 21,619 gene transcripts and approximately 1.3 million methylation sites remained, which were employed in further downstream analyses.

**Fig. 2:**
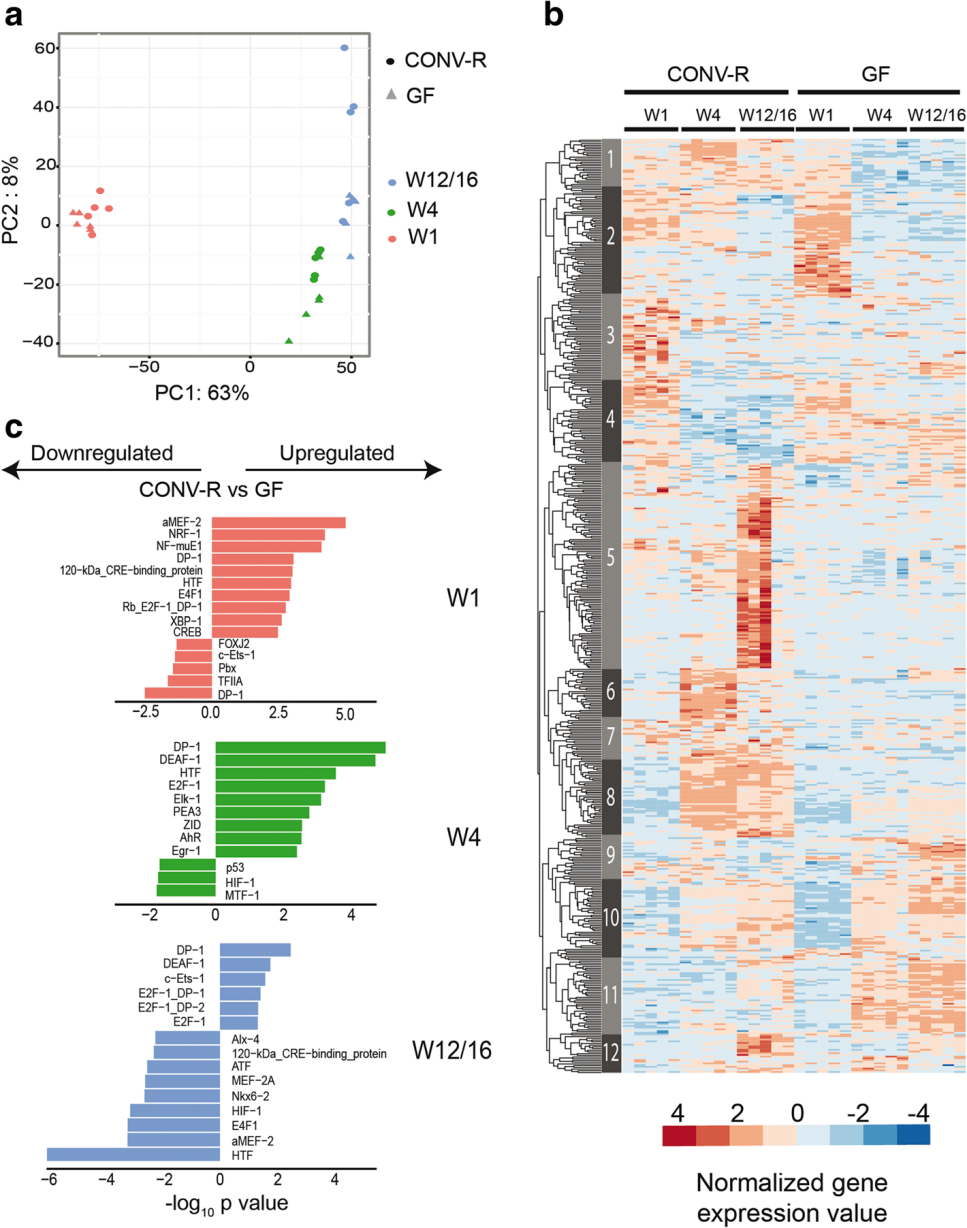
Microbial effects on the host epithelial transcriptome during postnatal development. **a** Principal component analysis displaying overall gene expression profiles across all samples. The first dimension explained 63% variation and separated W1 and the other two stages. The second dimension explained 8% variation and separated both W4 versus W12/16 and samples of a stage for their microbiota status. **b** Transcription factor binding sites enriched among microbially regulated genes (differentially expressed in CONV-R vs GF) for each of the three developmental stages. The bar plot depicts the 15 most significantly enriched transcription factors of either up- or downregulated genes. All *p* values were corrected for multiple testing using the Benjamini–Hochberg method. **c** Hierarchical clustering of microbially regulated genes identified 12 groups with specific expression profiles, e.g., group 3 genes that were repressed by the presence of the microbiota at W4 and W12/16 or conversely group 8 genes induced by the microbiota

First, we performed principal component analysis to visualize the global distribution of samples based on the expression data of the 21,619 transcripts. Samples were clustered according to both the developmental stage and microbial status (Fig. 2a). The first principal component explained 63% variation and separated samples from W1 and the other two stages, W4 and W12/16, indicating that gene expression changed dramatically during maturation of IECs, especially in the early postnatal period. The second principal component explained 8% of variation and separated W4 and W12/16 but also CONV-R and GF within a single developmental stage (Fig. 2a). Notably, the distance between CONV-R and GF samples increased along with time from W1 to W12/16. We detected 56 (0.3%) microbially regulated genes in W1 (differentially expressed in CONV-R vs GF comparison with BH-corrected *p* value < 0.05 and absolute fold change > 2), 614 (2.8%) in W4 and 1084 (5.0%) in W12/16 (Additional files 3 and 4). Moreover, the expression differences between CONV-R and GF (fold change) of the microbially regulated genes also increased with time (Additional files 3 and 5). Thus, ontogeny (developmental stage) and to a lesser extent bacterial status determine the epithelial transcriptional profile during postnatal development.

To gain insights into the biological functions of the microbially regulated genes during postnatal development, we employed Gene Ontology (GO) enrichment analysis on the differentially expressed genes in the three developmental stages. Supporting previous publications, enriched GO terms included mainly immune response-related or metabolic functions (Additional file 6).

We also tested whether postnatal and microbial status affected alternative splicing events. Overall, distribution of the splicing events did not differ significantly between CONV-R and GF mice or among the three developmental stages (Chi-squared test, *p* value = 0.99; Additional file 7). However, few distinct signatures were detectable that differentiated CONV-R from GF mice; for example, a higher number of microbiota-dependent intron retention events (2.3-fold higher, BH-corrected *p* value = 0.006, Chi-squared test with Yates continuity correction) in W1 compared to W4 or W12/16 (Additional files 7 and 8).

Next, we employed transcription factor binding site enrichment analysis among the promoters of microbially regulated genes to investigate the regulatory networks that underlie the microbiota-induced transcriptome alterations [58]. Interestingly, the transcriptional regulators most enriched among promoters of microbially regulated genes were unique to W1 whereas W4 and W12/16 shared several transcription factors (Fig. 2b). For example, in W1 the motif of the transcription factor XBP1, which functions in endoplasmic reticulum stress, cellular proliferation, and differentiation and protects from intestinal inflammation [59–61], was enriched in the promoters of genes upregulated by the microbiota. In W4 and W12/16 sites predicted to bind the transcription factor HIF1, which functions in mediating hypoxia effects and regulates metabolism and immune responses [62–64], were overrepresented among downregulated genes.

To identify co-regulated patterns of transcripts modulated by the microbiota we selected the 200 most significant genes regulated by microbial state at each of the three developmental stages, created the union of these genes (*n* = 547 genes), and performed hierarchical clustering analysis (depicted in the heatmap graph in Fig. 2c). A similar analysis was performed based on the selection of developmentally regulated genes for the two bacterial conditions CONV-R and GF (*n* = 553 genes; Additional file 9). The analyses revealed both a microbial imprint (e.g., clusters 2, 3, 4, 8, 11 in Fig. 2c) as well as a developmental effect (e.g., clusters 8, 10 in Fig. 2c) irrespective of the presence of bacteria. However, while the impact of postnatal development stage is clearly detectable in the visualization of microbially regulated genes (Fig. 2c), the influence of the presence of microbiota is less pronounced in the signature of the developmentally regulated genes. These data therefore support the previous finding that endogenous ontogenetic programs have a larger impact on the epithelial transcriptome compared with environmental cues from the commensal microbiota. Cluster 8 contains microbially responsive genes that mainly have functions in immune responses and are induced by the microbiota and the effect increases during development (Fig. 2c). Notably, genes of this cluster include *Duox2* (dual oxidase 2), *Reg3g* (regenerating islet-derived protein 3 gamma), *Nos2* (inducible nitric oxide synthase), *Saa1* (serum amyloid A-1), and *Saa2*, which have been reported previously as microbially induced in IECs [6]. The clusters 3 and 4 contain genes such as *Sdr16c6* (short chain dehydrogenase/reductase family 16C, member 6) or *Fn3k* (fructosamine 3 kinase), which are associated with metabolic functions, and expression of these genes increased specifically during W1 in colonized mice and then returned to basal level (Fig. 2c).

**Fig. 3:**
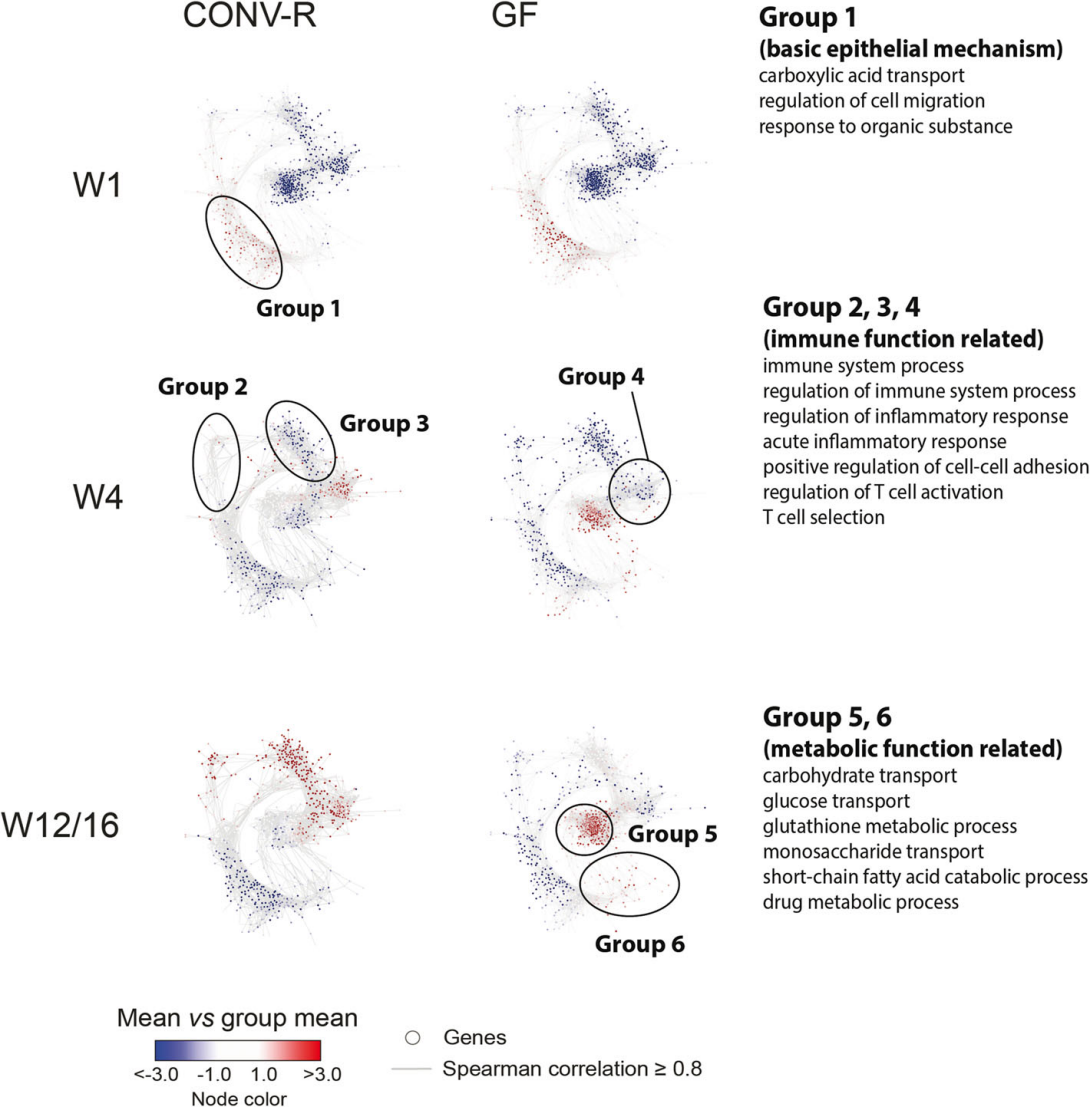
The microbiota modulates distinct functional expression nodes during postnatal development. Co-expression network analysis (CENA) was performed based on 970 co-expressed genes (correlation factor greater than 0.8 across all conditions). Each *dot* represents a gene and the color indicates its expression compared to the average gene expression level (*red* = up, *blue* = down). Note that ellipsoids represent only estimated visualization of transcript groups (for details see the “Methods” section). Exemplary GO terms enriched among the groups of co-regulated genes are listed, representing the main biological function of that gene group (for full list of GO terms see Additional file 10)

Next, we investigated the influence of the intestinal microbiota during postnatal development by co-expression network analysis [46, 65]. Co-expression network analysis builds on the hypothesis that genes with similar expression patterns are likely to have a functional relationship [66]. Following the procedure from Xue and colleagues [46], 970 co-expressed genes were selected based on a correlation cutoff of 0.8, normalized by their transcription level and tested for up- or downregulation compared to the average expression in the dataset (Additional file 4). Gene set enrichment analysis was used to identify the biological processes of individual time- and state-dependent co-expression subnetworks (Fig. 3, Additional file 10). At the W1 stage, we did not detect a prominent microbiota-dependent gene cluster (CONV-R and GF), but differential gene expression was exclusively time-dependent (W1 vs W4 vs W12/16, group 1). Genes of this group 1 were involved in basic epithelial maintenance. At the later postnatal stages W4 and W12/16 two compensatory microbiota-dependent transcriptional responses were evident. Several genes involved in immune function (groups 2, 3, and 4)—for example, *Duox2* (dual oxidase 2), *Nod2* (nucleotide-binding oligomerization domain containing 2), *Fut2* (fucosyltransferase 2), *Pigr* (polymeric immunoglobulin receptor), *Nos2* (nitric oxide synthase 2), or *Reg3g* (Regenerating islet-derived protein 3-gamma), which are expressed by IECs—were upregulated in CONV-R compared to GF mice, whereas genes encoding metabolic functions (groups 5 and 6)—for example, *Ces1d* (carboxylesterase 1D), *Pnliprp2* (pancreatic lipase-related protein 2), and *Slc5a4b* (solute carrier family 5, neutral amino acid transporters system A, member 4b)—were downregulated in CONV-R mice.

### Endogenous developmental programs as well as bacterial environmental cues affect the DNA methylation profile

**Fig. 4:**
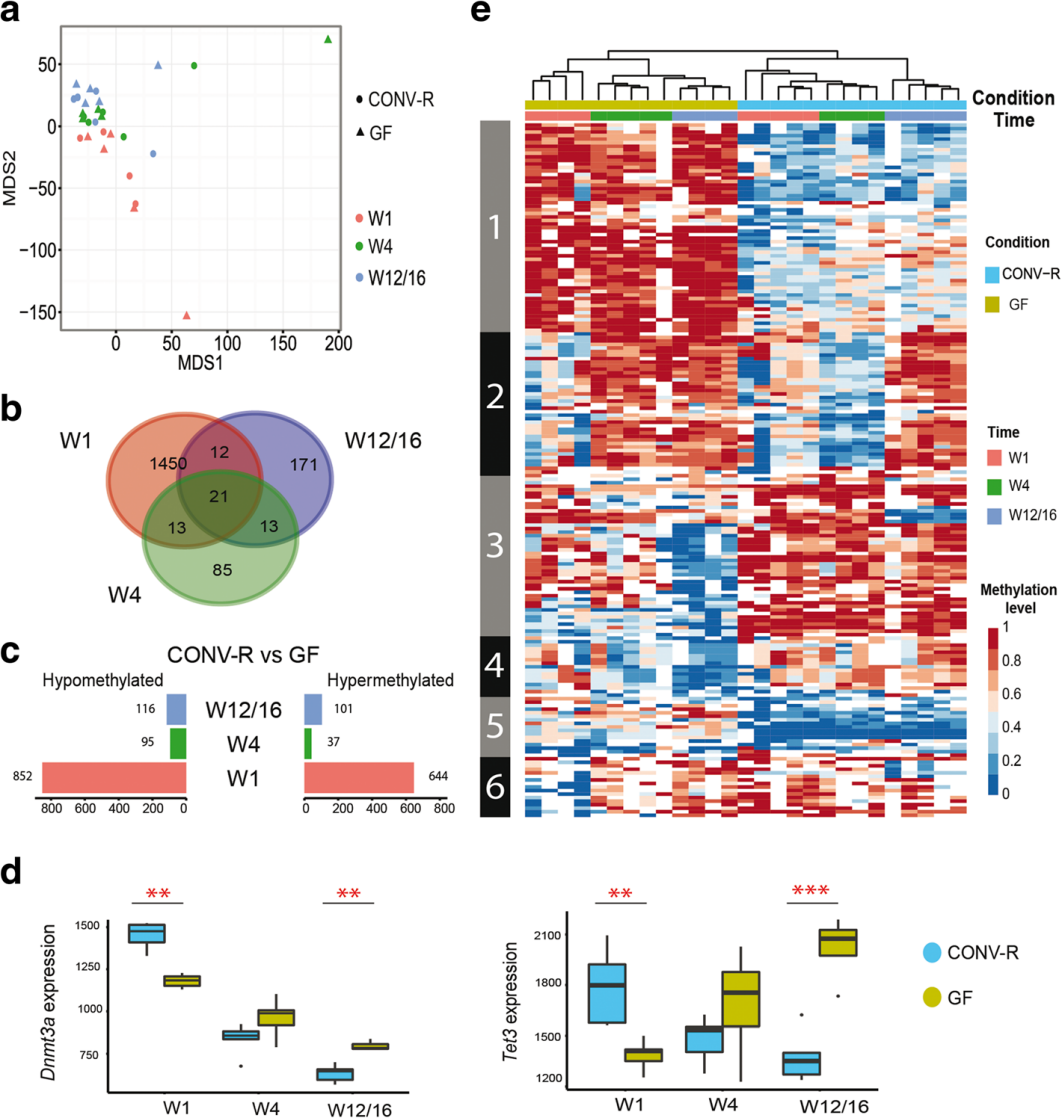
Postnatal development and the microbiota affect the DNA methylation profile. **a** Multidimensional scaling analysis plot displaying the overall methylation profiles. **b** Venn plots showing the number of differentially methylated sites between CONV-R and GF at the three developmental stages. Note the high number of differentially methylated sites at W1. **c** Number of hypo- and hypomethylated sites among all DMPs (CONV-R vs GF) for each developmental stage. **d** Expression of *Dnmt3a* and *Tet3* genes, which function in de novo methylation and demethylation, respectively. **e** Hierarchical clustering of differentially methylated sites between CONV-R and GF in the three developmental stages. Each row indicates a CpG site and the color scale represents the methylation level

To investigate how postnatal development and the microbial environment act on the DNA methylation pattern of IECs, we employed RRBS to determine the methylation level of isolated IECs from CONV-R and GF mice at W1, W4 and W12/16 (the identical samples used for transcriptome analysis). First, we examined the overall methylome pattern (1,296,536 CpG sites) by using multidimensional scaling analysis [67] instead of principal component analysis (PCA) due to data structure (“zero” inflation problem in RRBS as not all methylation sites can be detected in every sample regardless of sequencing depth). As for the transcriptome analysis, samples separated according to the developmental stage (Fig. 4a) and the methylation level increased with time (Additional file 11), indicating a strong effect of postnatal developmental programs on DNA methylation. In contrast to transcriptional signatures, the global scaling analysis did not reveal a strong effect of the microbiota on the overall DNA methylation pattern. By individual comparison of the DNA methylome of CONV-R and GF at each time point, however, we were able to identify 1496, 132, and 217 DMPs (FDR < 0.05) in W1, W4, and W12/16, respectively (Fig. 4b). Interestingly, the number of DMPs at the earliest stage was about 10× higher compared to that of the later stages, indicating that the microbiota acted stronger on DNA methylation during W1 or that the microbial state already acts in utero. Detected DMPs were equally hypo- and hypermethylated (Fig. 4c). We classified the relative position of the variant sites according to their genomic location as exonic, intronic, intergenic, or promoter-associated DMPs. Notably, in W1 DMPs located in gene promoter regions were enriched (175 DMPs or 11.7%) compared to W4 (one DMP or 0.8%) and W12/16 (15 DMPs or 6.9%) (Additional file 12). Given the enrichment of DMPs specifically during early development, we surveyed the expression of genes which are known to alter DNA methylation for microbial effects (Fig. 4d and Additional file 13). Expression of *Dnmt3a* and *Tet3* (Tet methylcytosine dioxygenase 3) were significantly altered by the microbiota in W1 and W12/16. DNMT3A is important for de novo methylation [68], whereas TET3 is essential for demethylation [69]. Similar to the approach of the transcriptome analysis, we ranked all DMPs based on their BH-corrected *p* value and chose the top 100 most significantly regulated DMPs from the microbiota-associated data set (Fig. 4e and Additional file 14) and from the developmental program (Additional file 15) for each time point to visualize differential methylation by hierarchical clustering. We chose a ranked approach and the top 100 to generate equal sample sizes for the analysis based on the total number of differentially methylated sites in the respective comparisons (minimum 132 for W4). For the microbiota-related DMPs, samples clustered according to microbial status and developmental stage (Fig. 4e) except for a few samples with several missing values only among these microbiota-related DMPs, which may be due to insufficient sequencing depth. However, these samples did contain data for many other of the almost 1.2 million CpG sites. As the samples overall met the quality criteria, they were not removed from the methylome analysis. For the top 100 developmentally related DMPs at each time point, samples clustered only by developmental stage but did not reveal a further stratification according to microbial status (Additional file 15).

### Integrated analysis identifies a specific signature of loci with coupled DNA methylation and RNA transcription driven by the presence of microbiota

**Fig. 5:**
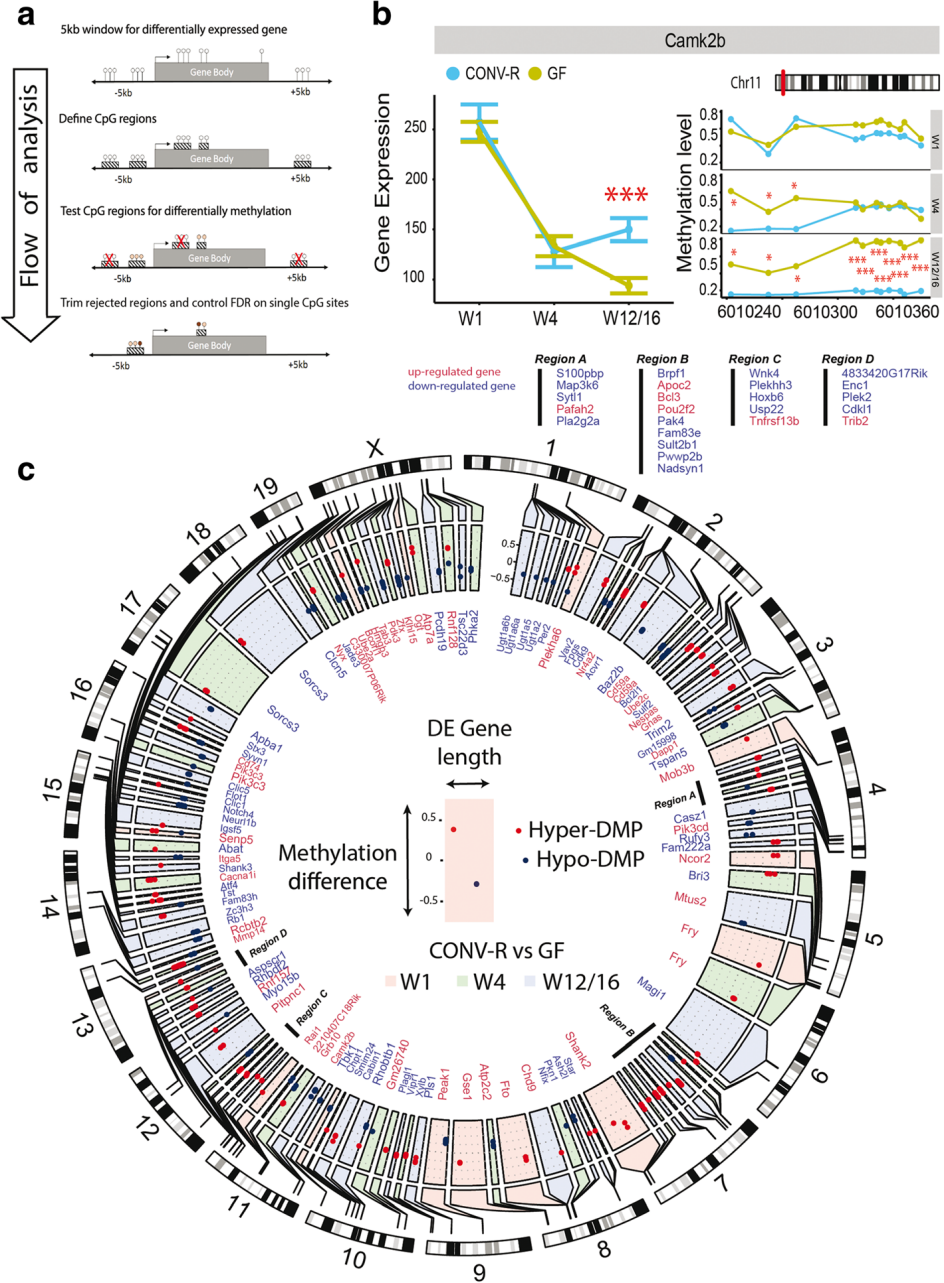
The microbiota may modulate host gene expression through DNA methylation. **a** Schematic analysis workflow. A 5-kb window up- and downstream of each microbially regulated gene was screened for CpG positions. Next, CpG regions were defined and tested for differential methylation (CONV-R vs GF) and *p* values of all differentially methylated CpG sites were corrected for multiple testing. It is noteworthy that any sequential analysis reflects a certain bias by the individual order of filter steps. **b** Microbial effects on gene expression and DNA methylation of *Camk2b* during postnatal development. **c** Genomic map of all methylation–transcription interactions dependent on the microbiota and postnatal development. The *boxes* in the *outer circle* depict the mouse chromosomes and their banding indicates the staining properties within the genomic locations (*black* = heterochromatin region, *white* = euchromatin region, *gray* = intermediate). The *boxes* in the inner circle represent genes that were both differentially expressed and methylated. The gene name is colored according to the expression difference in CONV-R vs GF comparison (*red* = upregulated, *blue* = downregulated). Box coloring corresponds to the developmental stage, in which a significant difference was detected (*red* = W1, *green* = W4, *blue* = W12/16). Width of the boxes indicates gene length, while methylation differences in CONV-R vs GF comparison are scaled along the height of the boxes. *Red* and *blue dots* within the gene boxes represent hyper- and hypomethylated CpG sites, respectively

**Fig. 6:**
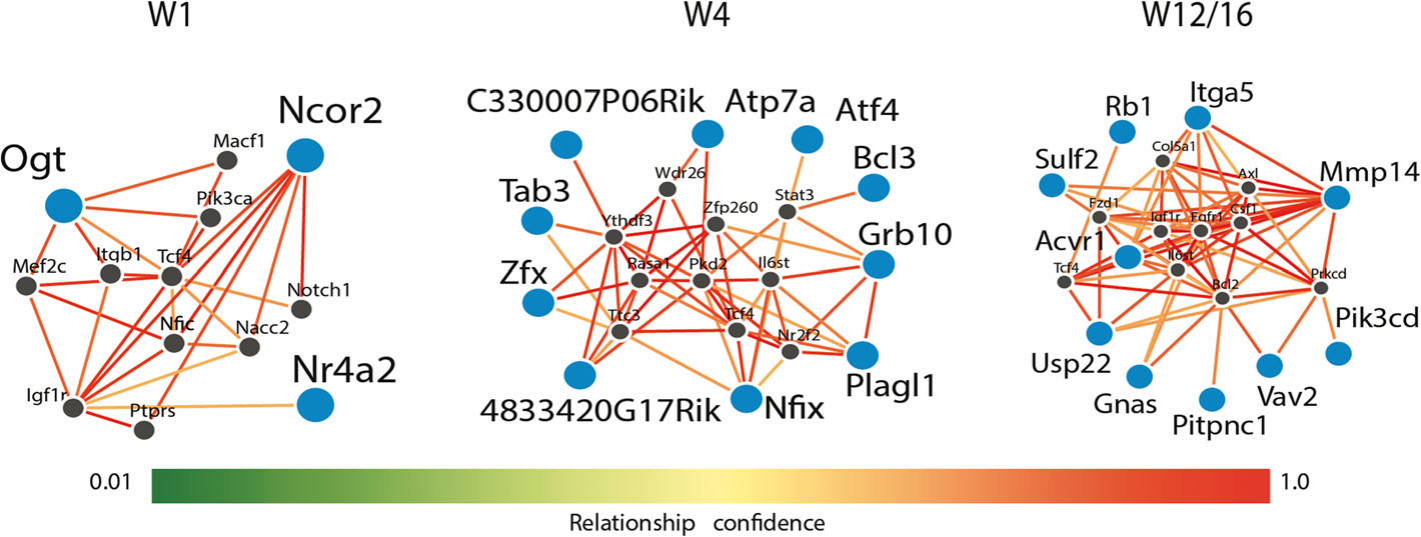
Integrated analysis identifies genomic loci with coupled differential DNA methylation and RNA transcription associated with the presence of intestinal microbiota. Network analysis based on differentially methylated and differentially expressed genes (CONV-R vs GF) across the three developmental stages with a relationship confidence greater than 0.6. *Larger blue circles* indicate candidate genes identified from our analysis and *smaller black circles* denote imputed interacting genes

Next, we sought to identify microbiota-dependent DNA methylation changes linked to RNA expression differences. We hypothesized that this mode of regulation may pinpoint important genes involved in epithelial–microbe interaction as it represents a potentially longer-term modulation of cellular programs. We employed a hierarchical testing approach [52] to identify interactions between the microbiota-dependent alterations in the transcriptome and DNA methylation signatures (Fig. 5a). To that end, we screened all differentially expressed genes (CONV-R vs GF) for DMPs within a 5-kb window up- and downstream. We identified 17, 34, and 79 microbially regulated genes both with altered expression and differentially methylated in W1, W4, and W12/16, respectively, and most (122 out of 126) were specific for the developmental stage (Additional files 16 and 17). Tracking both the transcriptome and DNA methylation in paired samples from individual mice throughout early postnatal development allowed us to identify specific changes in the DNA methylation signature that may underlie the microbiota-dependent transcriptome alterations. For example, expression of *Camk2b* (calcium/calmodulin-dependent protein kinase II), which is involved in calcium-dependent signaling [70], was only altered by the microbiota at W12/16 but not at the younger stages W1 or W4 (Fig. 5b). Interestingly, nearby CpG sites were not differentially methylated at W1, whereas in week W4 we detected three DMPs and another eight DMPs at W12/16 (Fig. 5b). Therefore, either the complete demethylation of all 11 DMPs or only the eight downstream DMPs may be required to mediate the microbial induction of *Camk2b* expression at W12/16. Similarly, *Mob3b* (MOB kinase activator 3B) and *Ube2a* (Ubiquitin conjugating enzyme E2 A) were differentially methylated and expressed only at W1 and W4, respectively, but not at any other developmental stage (Additional file 18). Of all 126 genes with differential expression and methylation 72 (57%) showed increased expression with reduced methylation or decreased expression with increased methylation, whereas 54 genes (43%) did not show a canonical association of expression and methylation shift, which is similar to previous studies [24]. Genome-wide mapping of the host–microbiota interactions for gene expression and DNA methylation during the three development stages revealed equal distribution among chromosomes (Fig. 5c). Among all genes that were differentially methylated and expressed depending on the microbiota, network analysis revealed an enrichment of genes involved in regulation of cellular proliferation and regeneration, such as *Pik3cd*, *Rb1*, *Grb10*, *Plagl1*, *Nfix*, and *Tab3*, or of genes functioning in immune responses, such as *Atp7a*, *Atf4*, and *Bcl3* (Fig. 6). For example, *Rb1* (retinoblastoma-associated protein) is a tumor suppressor inhibiting cell cycle progression, which may also recruit methylases [71]. *Rb1* expression was reduced in CONV-R mice, which is in line with an increased IEC proliferation in the presence of a microbiota [6, 9]. Similarly, *Bcl3* is a proto-oncogene promoting proliferation and also mediates immune tolerance by suppressing responses against microbial antigens [72]. In our analysis *Bcl3* was hypomethylated and expression increased in CONV-R mice, which is supported by a higher proliferative capacity in the presence of a microbiota. Finally, as another example, *Plagl1* (pleiomorphic adenoma gene-like 1), which is another tumor suppressor inhibiting proliferation, was hypomethylated and had higher transcript levels in CONV-R mice, again supporting increased IEC proliferation in the presence of a microbiota.

To validate our findings, we selected a subset of the differentially expressed and methylated genes from our data and determined their expression and DNA methylation in an independent cohort of GF and CONV-R mice from another gnotobiotic animal facility. We harvested small intestinal epithelial tissue by scraping, isolated RNA and DNA as before, and performed qPCR analysis along with amplicon sequencing. For the eight tested genes (*Bcl3, Nfix, Cacna1i, Rcbtb2, Mmp14, Itga5, Cd74*, and *Pik3cd*) differential expression and methylation was reproduced for six genes in both cases (Additional file 19).

## Discussion

We systematically investigated the regulatory effects of the microbiota on the transcriptome and the genome-wide DNA methylation status of IECs from the small intestine of infant, juvenile, and adult mice which were raised in either the presence or absence of a microbiota. This analysis revealed that both the IEC ontogeny and the microbiota affect the epithelial transcriptome signature along with the DNA methylation status and that the microbial effect increases during postnatal development. Furthermore, the microbial impact on the interplay of DNA methylation and the epithelial transcriptome were stage-specific as we detected almost no overlap between the genes that were regulated by the microbiota and also displayed an altered DNA methylation status for the three developmental stages. Our data provide groundwork to further dissect the endogenous developmental and microbial effects on the host’s transcriptional and epigenetic program on a mechanistic level.

To fully understand the impact and role of the microbiota during adult development of IECs, it is required to assess the transcriptional and epigenetic changes over time in both GF and CONV-R animals with a large enough size of biological replicates. While several studies have addressed selected aspects of the interplay of transcription, epigenetics, development, and microbiota [6–10, 12, 18, 29, 73, 74], an integrated genome-wide analysis of DNA methylation and transcriptional signatures in a single study using biological replicates and animals from different GF colonies has so far been lacking. In our current study, we therefore determined the epigenetic and transcriptional interactions between the gut microbiota and IECs using an integrated analysis of the methylome and transcriptome over time in both GF and CONV-R mice. The value of our experimental approach is demonstrated by the finding that although several previous studies established that the microbiota modulates the expression of more than 2000 genes in the intestinal epithelium [6, 9, 10], only a subset of these microbiota-responsive genes appear to be regulated by the epigenetic process of DNA methylation. Using our approach, we found that the microbiota seemed to inversely affect DNA methylation and gene expression throughout postnatal development. Whereas the number of differentially expressed (CONV-R vs GF) genes increased with postnatal development, the number of DMPs decreased from W1 to W12/16. The number of genes for which both transcription and DNA methylation are regulated by the microbiota (differentially expressed and DMPs within a 5-kb window) increased with time. Together these observations indicate that the microbial effect on modifying the epithelial DNA methylation and transcriptional status increased during maturation and postnatal development of the intestine. Notably, W1 samples differed substantially from W4 and W12/16 samples, indicating that further studies are required to describe the early dynamics from W1 to W4 in greater detail. However, the microbiota did not seem to engage DNA methylation to regulate transcriptional responses globally, but instead only seemed to target a specific subset of microbially responsive genes through their DNA methylation status. This unexpected finding is not caused by inherent differences in our and published datasets as, for example, our transcriptome sequencing data and the list of microbially regulated genes from the adult stage overlapped significantly with our previous data obtained from microarray analysis of laser-dissected ileal IECs [6]. Our observations are further supported by a study by Camp et al. which reported that the microbiota did not globally alter the chromatin architecture to drive gene expression, but only for specific genes [12]. Thus, host epigenetic mechanisms do not seem to be employed by the gut microbiota to drive transcriptional responses on a global scale.

Our study further validated that many developmentally regulated genes such as *Pigr*, which was reported to have increasing expression from infant to juvenile, or *Tet1*, having a decreasing expression from infant to juvenile [73], in addition also were differentially methylated and therefore appeared to be epigenetically regulated during postnatal development. Moreover, we could show that several of the genes which were previously reported as microbially regulated in the adult [6, 10] were also regulated transcriptionally during postnatal development. For example, the glycolysis regulator *Pfkfb3* (6-phosphofructo-2-kinase) was not only induced by the microbiota in the adult as reported [6, 10], but is already microbially regulated in the infant.

Surprisingly, we detected about ten times more DMPs in W1 compared to W4 or W12/16. Since methylation levels did not differ between the developmental stages, the increased number of DMPs in W1 did not seem to be simply due to higher overall methylation activity. Instead, the microbiota may differentially modulate de novo methylation and demethylation in the neonate mice. First, we detected generally higher levels of *Dnmt3a* during W1 compared to W4 or W12/16 and increased expression in CONV-R compared to GF mice. As DNMT3 mediates de novo methylation and parental imprinting [75], this temporal and microbiota-dependent expression pattern of *Dnmt3a* may therefore relate to the increased number of hypermethylated DMPs in the newborn mice. Conversely, *Tet3* expression was induced by the microbiota in W1 and since TET3 possesses hydroxymethylation activity [76, 77] and therefore mediates demethylation [69], the time- and microbiota-dependent expression pattern of *Tet3* may thus contribute to the increasing number of hypomethylated DMPs with increasing age. However, we can also not rule out a maternal imprinting effect, which may be dependent on the presence of microbiota in the mother before birth. Since the two groups of mice (CONV-R and GF) in the discovery cohort represent two separate colonies originating from different multiple mothers, we cannot exclude differential transgenerational inheritance of selected methylation marks (from the mother to the pups). In addition, as GF and CONV-R mice have been maintained separately for several generations, genetic drift occurring in the two mouse colonies could theoretically contribute to the observed signatures, as genetic variants may have affected methylation sites. However, we validated a selection of identified differentially methylated and differentially expressed genes in an independent cohort of mice from another colony from a different gnotobiotic animal facility (Max-Planck Institute, Plön) using qPCR and targeted amplicon sequencing of the DMP loci. The validation of several candidate genes in an independent cohort—although of a smaller scale—corroborates the existence of microbiota-induced “functional” methylation sites, which may impact on long-term gene expression signatures in IECs.

Future studies are needed to functionally validate the involvement of methylation-modifying enzymes during early postnatal development and in relation to the microbiota. For example, tracking the changes in intestinal microbiota composition along with epithelial DNA methylation and transcriptome signatures of DNMT- or TET-deficient mice during postnatal development would be a promising approach. Together our data suggest that the microbiota seems to engage components of the DNA methylation machinery, which may at least partially translate into the observed epigenetic and transcriptional differences through postnatal development.

## Conclusions

Postnatal development affects DNA methylation signatures and expression in intestinal epithelial cells, indicating that epigenetic processes contribute to developmental transitions largely driven by endogenous programs independent of microbial cues. However, our data also clearly show that the gut microbiota influences specific modules of the epithelial transcriptional network during postnatal development and targets only a subset of microbially responsive genes mainly functioning in IEC proliferation and immune responses through their DNA methylation status.

### Additional files

Additional file 1: List of primers used for validation experiment (qPCR and amplicon sequencing). (XLSX 49 kb)

Additional file 2: R code used for the integrated analysis shown in Fig. 5a. (R 1 kb)

Additional file 3: Venn diagram of differentially expressed genes (CONV-R versus GF, adjusted *p* value < 0.05, fold change > 2) in the three developmental stages. (PDF 409 kb)

Additional file 4: Gene expression data of small intestinal epithelial cells from germ-free (GF) and conventionally raised (CONV-R) mice at the three developmental stages W1, W4, and W12/16 determined by RNA sequencing. (XLSX 9442 kb)

Additional file 5: MA transcriptome plot for CONV-R versus GF comparison. Every *dot* represents one transcript. The x-axis denotes the mean expression value and the y-axis denotes the log2 fold change of CONV-R versus GF. *Red dots* indicate statistically significant transcripts (CONV-R versus GF, adjusted *p* value < 0.05). The ceiling/floor of two on log2 fold change (y-axis) is set because of better visualization. (PDF 632 kb)

Additional file 6: Gene ontology (GO) analysis of the microbially regulated genes from each developmental stage. (XLSX 39 kb)

Additional file 7: Alternative splicing analysis. **a** Overview of the five categories of alternative splicing (skipped exon, alternative 5′ splice site, alternative 3′ splice site, mutually exclusive exons, and retained intron) as analyzed by the rMATS program. **b** Pie charts of the relative composition of alternative splicing events in each sample group. The relative composition patterns of alternative splicing do not differ significantly among the groups. **c** Count of significantly different (CONV-R versus GF, *p* < 0.05) alternative splicing events in the five categories for each developmental stage. The number of retained intron events in W1 was significantly higher than in the other stages. (PDF 529 kb)

Additional file 8: Alternative splicing events (total and only significant events in CONV-R versus GF) in the three developmental stages. (XLSX 9 kb)

Additional file 9: Heatmap of developmentally regulated genes (*n* = 553 genes). (PDF 818 kb)

Additional file 10: Gene ontology (GO) analysis of the co-expressed genes in different selected groups from Fig. 3. (XLSX 53 kb)

Additional file 11: Methylation levels across all samples (median ± standard deviation). (PDF 384 kb)

Additional file 12: Genomic location of DMPs (CONV-R versus GF) in the three developmental stages. (PDF 384 kb)

Additional file 13: Expression analysis of selected genes involved in DNA methylation: *Dnmt1* (DNA methyltransferase 1), *Dnmt3b* (DNA methyltransferase 3b), *Tet1* (Tet methylcytosine dioxygenase 1), *Tet2* (Tet methylcytosine dioxygenase 2), *Uhrf1* (Ubiquitin-like containing PHD and RING finger domains 1), *Uhrf2* (Ubiquitin-like containing PHD and RING finger domains 2), *Mbd2* (Methyl-CpG Binding Domain Protein 2), *Mbd3* (Methyl-CpG Binding Domain Protein 3), *Foxo3* (Forkhead box O3). (PDF 448 kb)

Additional file 14: Methylation levels of microbiota- (CONV-R versus GF) and development-dependent (W1 versus W4 versus W12/16) DMPs. Hierarchical clustering resulted in ten DMP groups. (XLSX 203 kb)

Additional file 15: Heatmap of methylation levels for developmentally related methylation sites. (PDF 658 kb)

Additional file 16: Venn diagram of differentially expressed genes (CONV-R versus GF) that also contain DMPs within a 5-kb window. (PDF 377 kb)

Additional file 17: List of differentially expressed genes (CONV-R versus GF) that also contain DMPs as depicted in Additional file 13. (XLSX 45 kb)

Additional file 18: Gene expression and DNA methylation levels of *Mob3b* (MOB kinase activator 3B) and *Ube2a* (Ubiquitin conjugating enzyme E2 A) genes and genomic loci in CONV-R and GF mice during postnatal development. (PDF 438 kb)

Additional file 19: Validation of a subset of differentially expressed and methylated genes. Small intestinal epithelial tissue was harvested by scraping from an independent cohort of GF and CONV-R mice from another gnotobiotic animal facility and both DNA and RNA were isolated for qPCR expression analysis and targeted methylation analysis using amplicon sequencing. *Asterisks* denote observations in the validation data that showed the same trend/direction as in the initial data, but were only very close to reaching the significance threshold after correction for multiple testing and therefore were considered as validation. (XLSX 48 kb)

## Abbreviations

CONV-R: conventionally raised specific pathogen-free
CpG: DNA motif with cytosine followed by a guanine
DMP: differentially methylated position
DNMT: DNA methyltransferase
DTT: dithiothreitol
EDTA: ethylenediaminetetraacetic acid
FDR: false discovery rate
GF: germ-free
GO: Gene Ontology
HDAC: histone deacetylase
IEC: intestinal epithelial cell
MDS: multidimensional scaling
PCA: principal component analysis
RNA-Seq: RNA sequencing
RRBS: reduced representation bisulfite sequencing
SCFA: short-chain fatty acids
W1, W4, W12/16: 1, 4 or 12/16 week-old mice
