# Supplementary figures and images for: Exposure to the gut microbiota drives distinct methylome and transcriptome changes in intestinal epithelial cells during postnatal development
Source: Genome Med. 2018 Apr 13;10:27. doi: 10.1186/s13073-018-0534-5 (PMC5899322; doi:10.1186/s13073-018-0534-5)

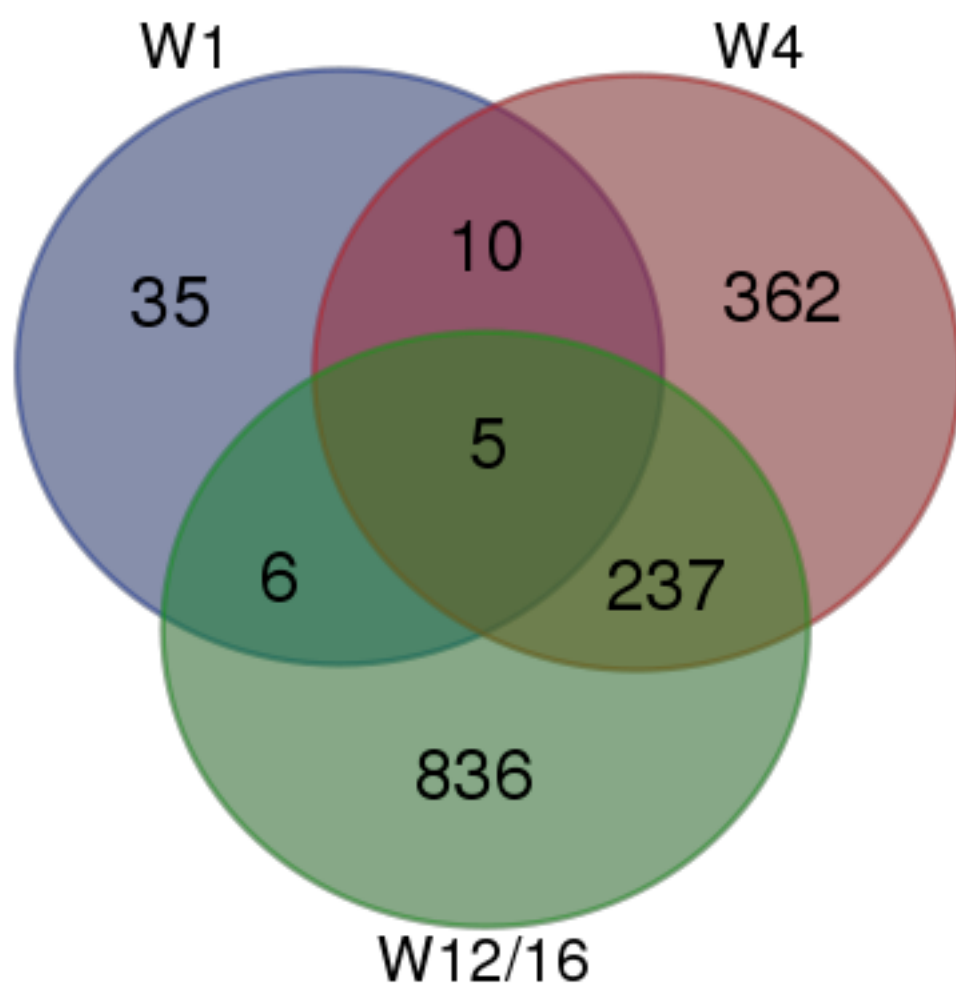

Supplement: Supplementary file 3 — Venn diagram of differentially expressed genes (CONV-R versus GF, adjusted p value < 0.05, fold change > 2) in the three developmental stages. (PDF 409 kb) [file 13073_2018_534_MOESM3_ESM.pdf]

W1

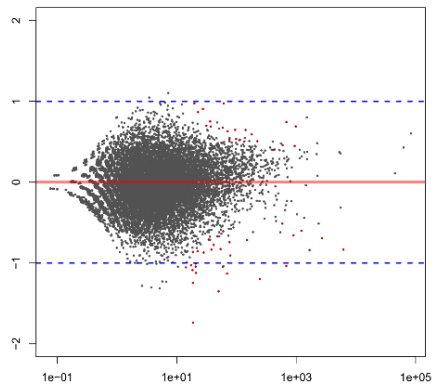

W4

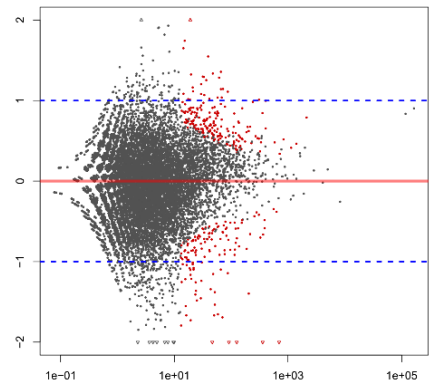

W12/16

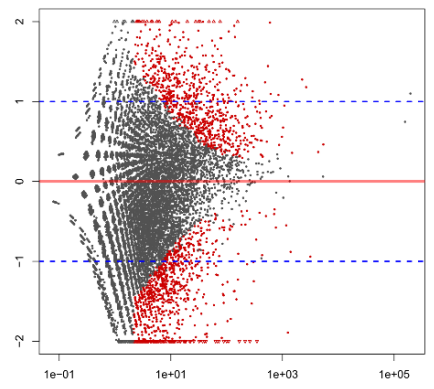

Supplement: Supplementary file 5 — MA transcriptome plot for CONV-R versus GF comparison. Every dot represents one transcript. The x-axis denotes the mean expression value and the y-axis denotes the log2 fold change of CONV-R versus GF. Red dots indicate statistically significant transcripts (CONV-R versus GF, adjusted p value < 0.05). The ceiling/floor of two on log2 fold change (y-axis) is set because of better visualization. (PDF 632 kb) [file 13073_2018_534_MOESM5_ESM.pdf]

A

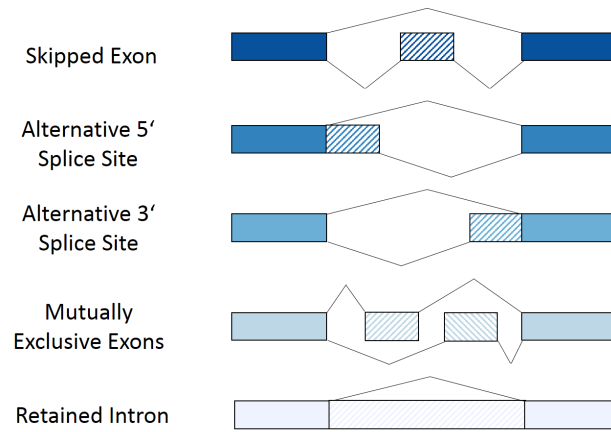

B

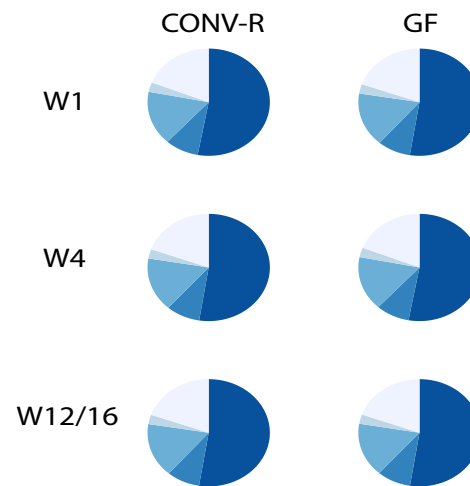

C

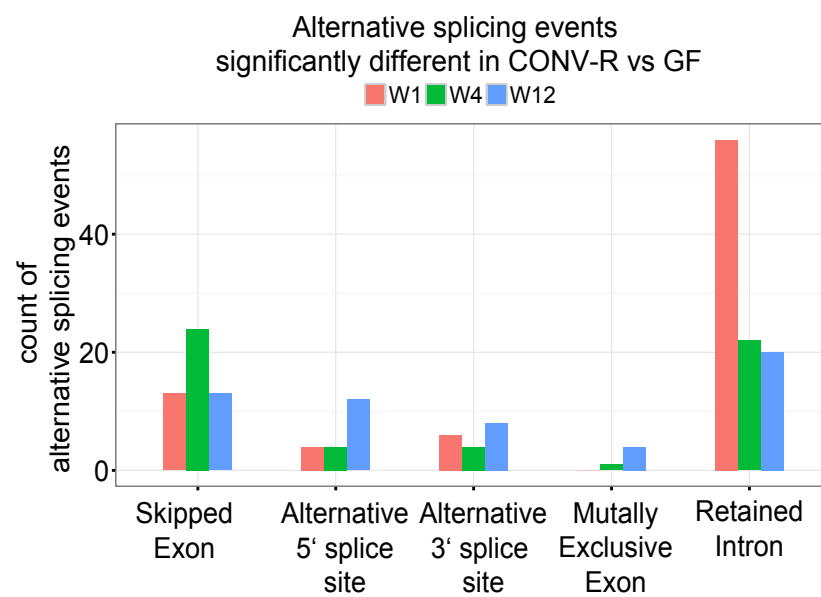

Supplement: Supplementary file 7 — Alternative splicing analysis. a Overview of the five categories of alternative splicing (skipped exon, alternative 5′ splice site, alternative 3′ splice site, mutually exclusive exons, and retained intron) as analyzed by the rMATS program. b Pie charts of the relative composition of alternative splicing events in each sample group. The relative composition patterns of alternative splicing do not differ significantly among the groups. c Count of significantly different (CONV-R versus GF, p < 0.05) alternative splicing events in the five categories for each developmental stage. The number of retained intron events in W1 was significantly higher than in the other stages. (PDF 529 kb) [file 13073_2018_534_MOESM7_ESM.pdf]

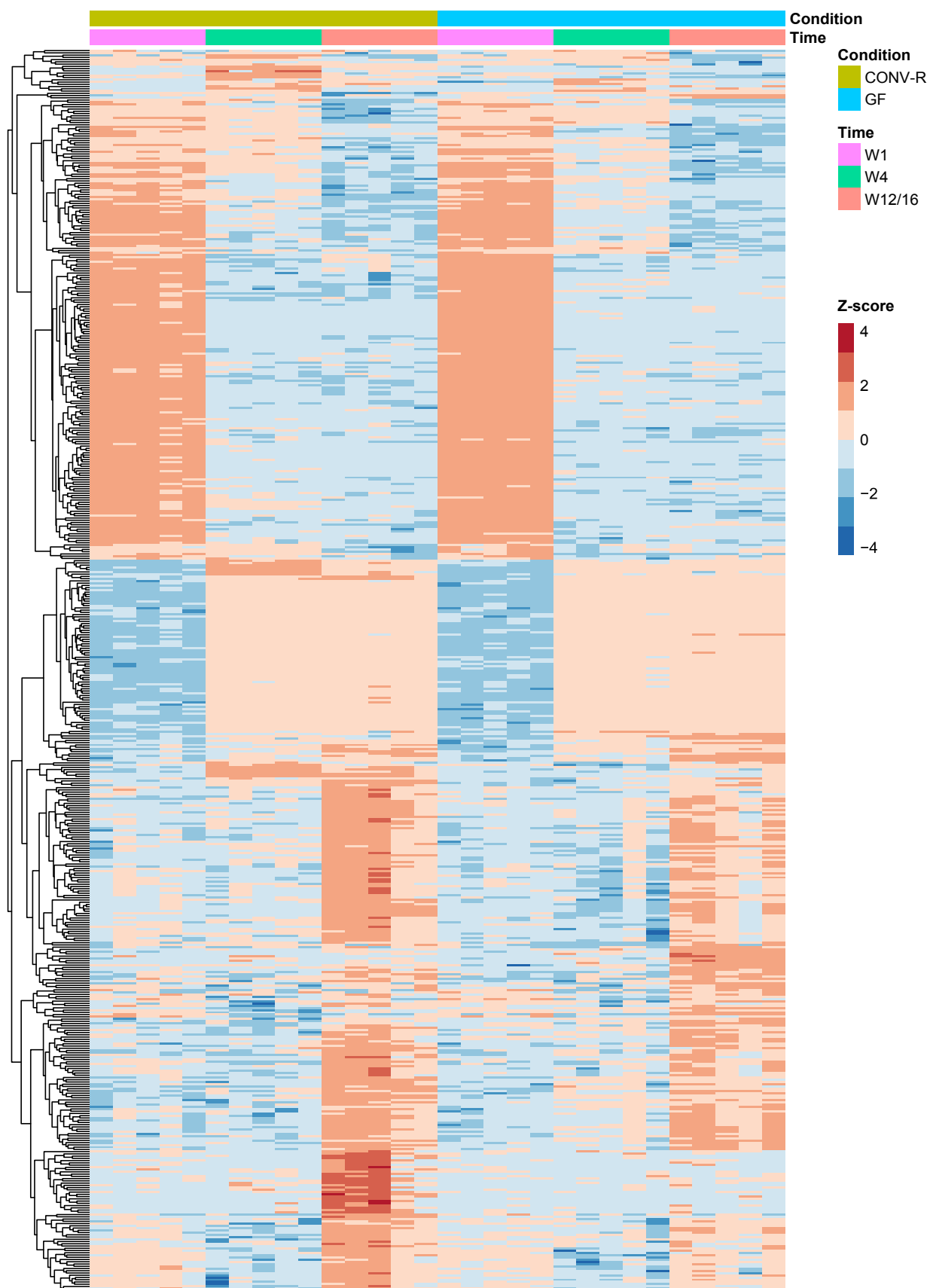

Supplement: Supplementary file 9 — Heatmap of developmentally regulated genes (n = 553 genes). (PDF 818 kb) [file 13073_2018_534_MOESM9_ESM.pdf]

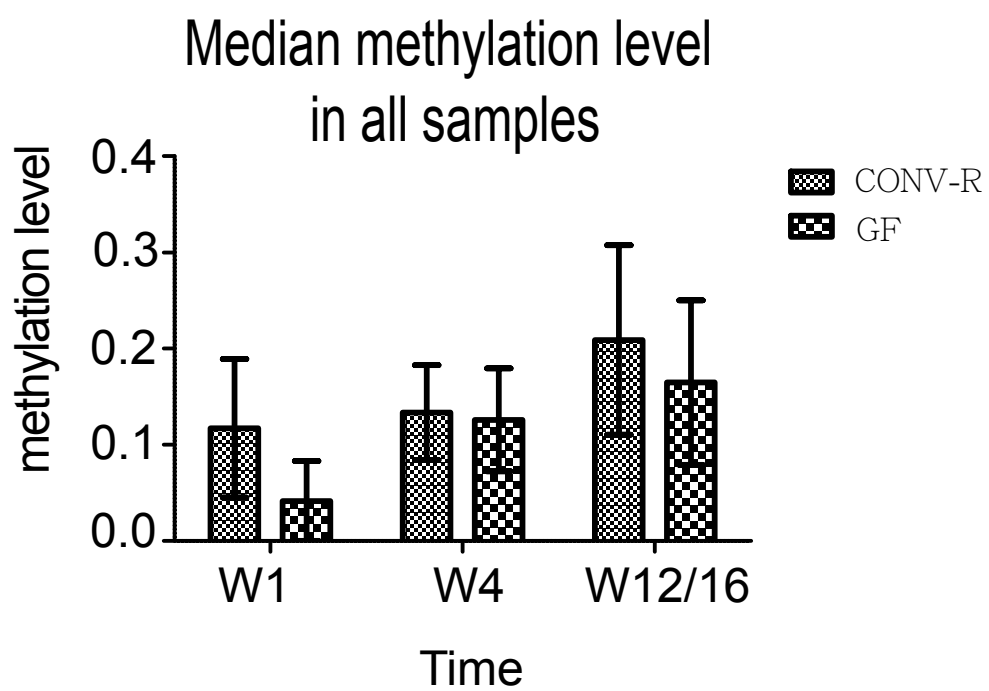

Supplement: Supplementary file 11 — Methylation levels across all samples (median ± standard deviation). (PDF 384 kb) [file 13073_2018_534_MOESM11_ESM.pdf]

**DMPs in W1**

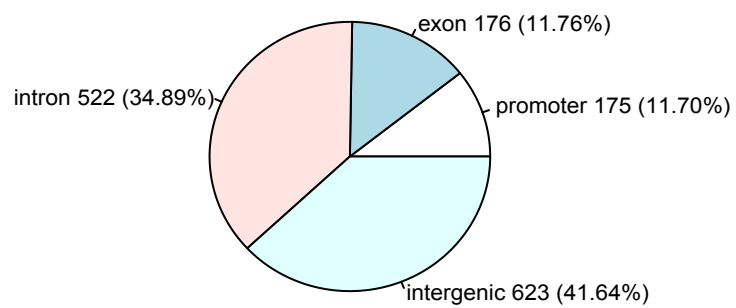

**DMPs in W4**

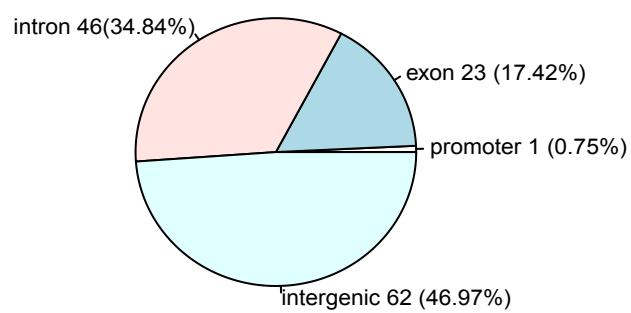

**DMPs in W12/16**

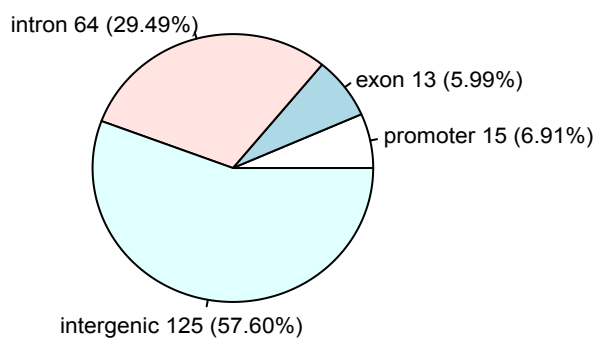

Supplement: Supplementary file 12 — Genomic location of DMPs (CONV-R versus GF) in the three developmental stages. (PDF 384 kb) [file 13073_2018_534_MOESM12_ESM.pdf]

● CONV-R ● GF

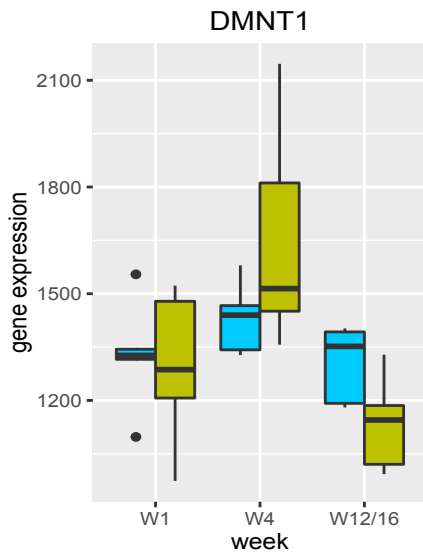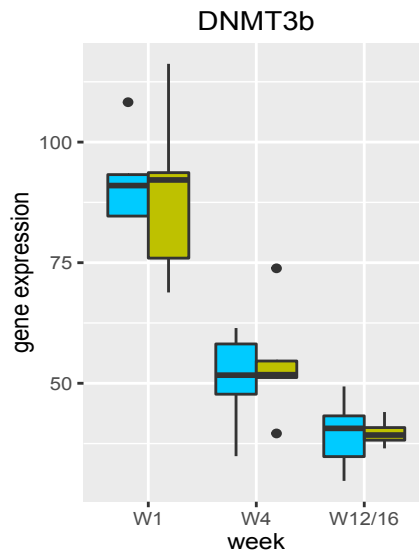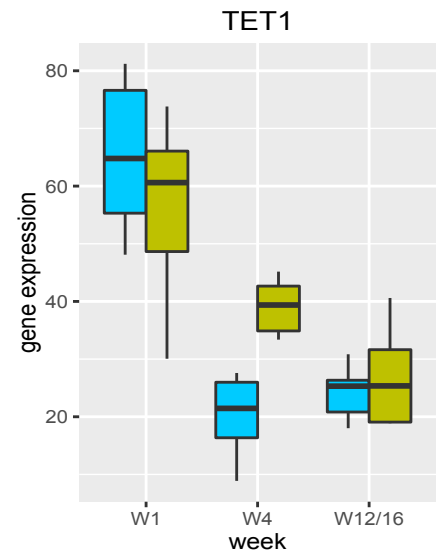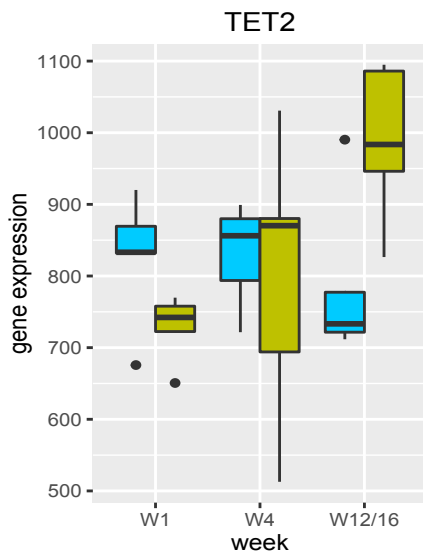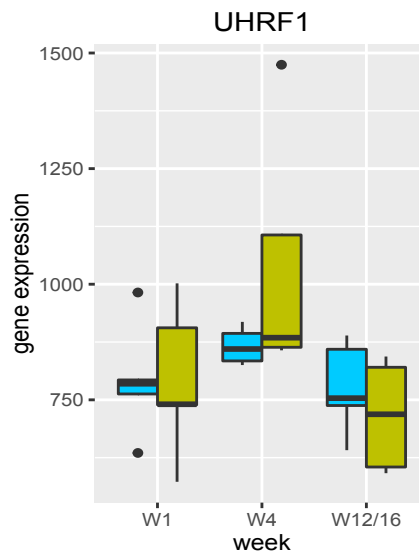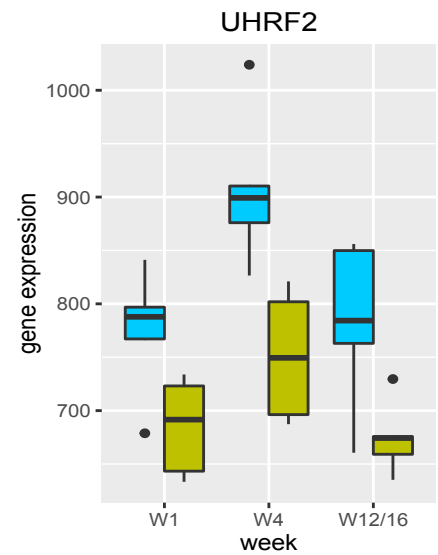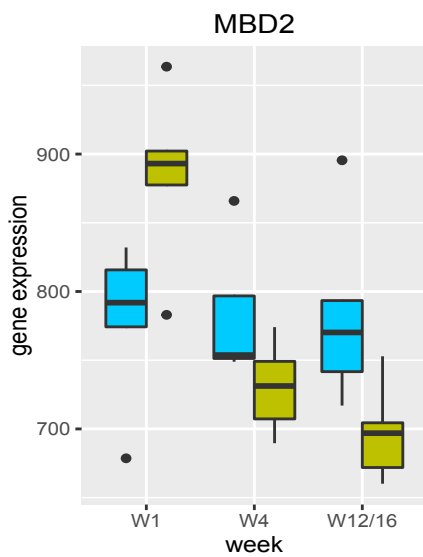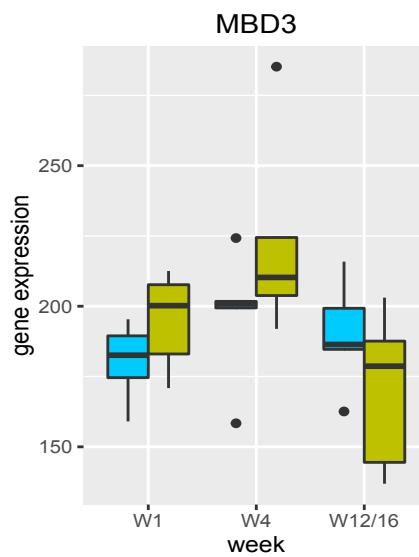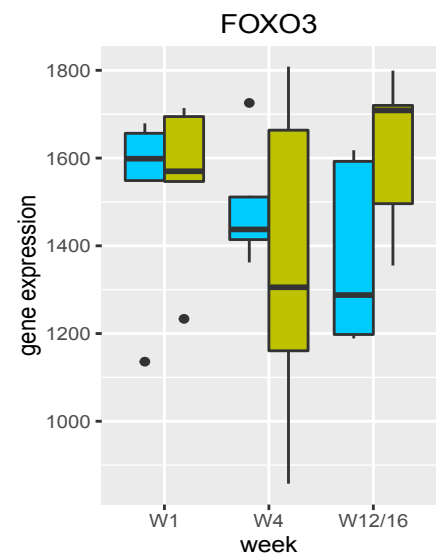

Supplement: Supplementary file 13 — Expression analysis of selected genes involved in DNA methylation: Dnmt1 (DNA methyltransferase 1), Dnmt3b (DNA methyltransferase 3b), Tet1 (Tet methylcytosine dioxygenase 1), Tet2 (Tet methylcytosine dioxygenase 2), Uhrf1 (Ubiquitin-like containing PHD and RING finger domains 1), Uhrf2 (Ubiquitin-like containing PHD and RING finger domains 2), Mbd2 (Methyl-CpG Binding Domain Protein 2), Mbd3 (Methyl-CpG Binding Domain Protein 3), Foxo3 (Forkhead box O3). (PDF 448 kb) [file 13073_2018_534_MOESM13_ESM.pdf]

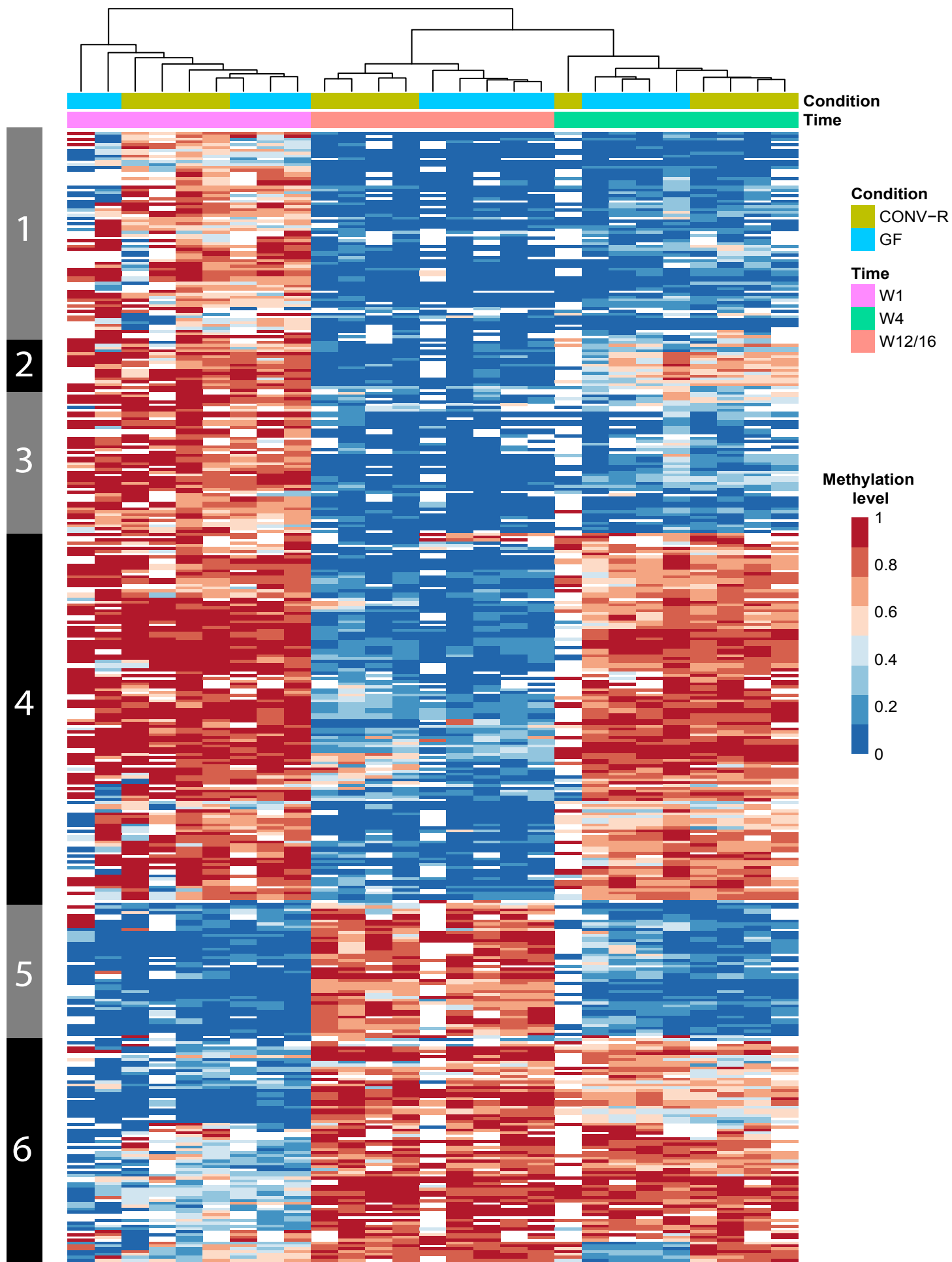

Supplement: Supplementary file 15 — Heatmap of methylation levels for developmentally related methylation sites. (PDF 658 kb) [file 13073_2018_534_MOESM15_ESM.pdf]

CD59a SORCS3 PIK3C3

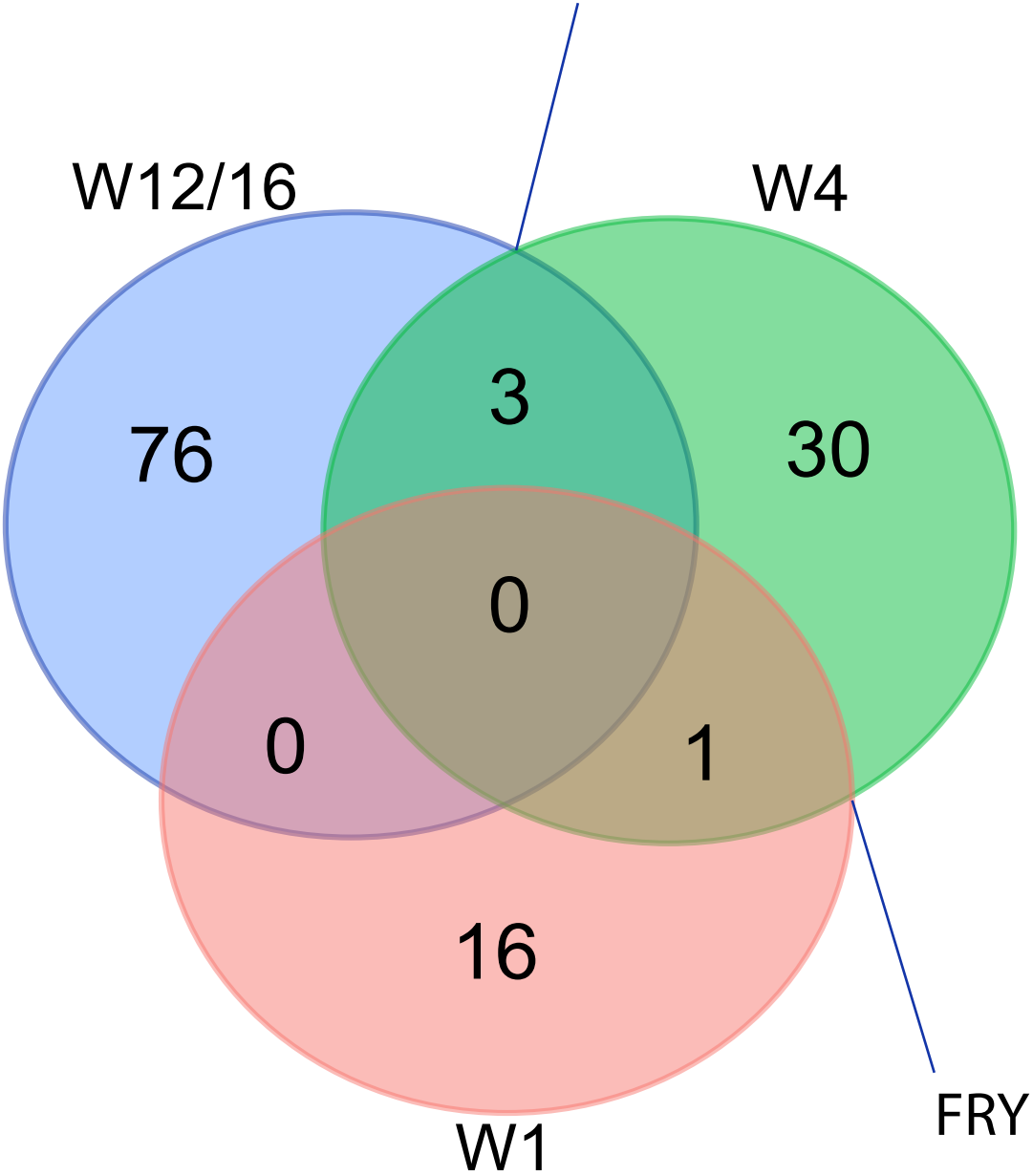

Supplement: Supplementary file 16 — Venn diagram of differentially expressed genes (CONV-R versus GF) that also contain DMPs within a 5-kb window. (PDF 377 kb) [file 13073_2018_534_MOESM16_ESM.pdf]

## Mob3b

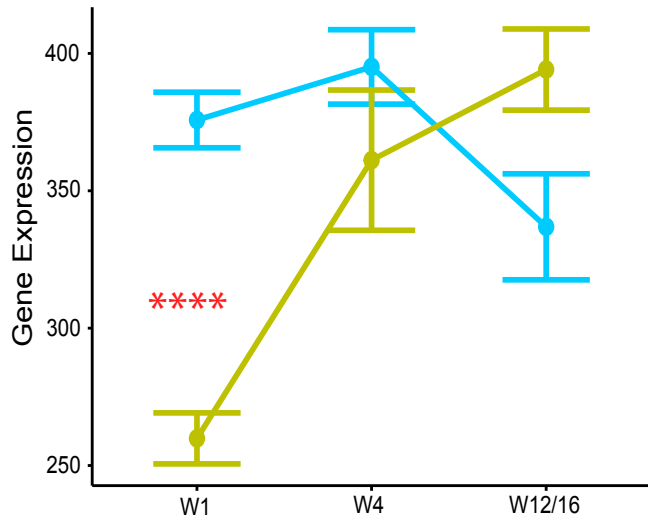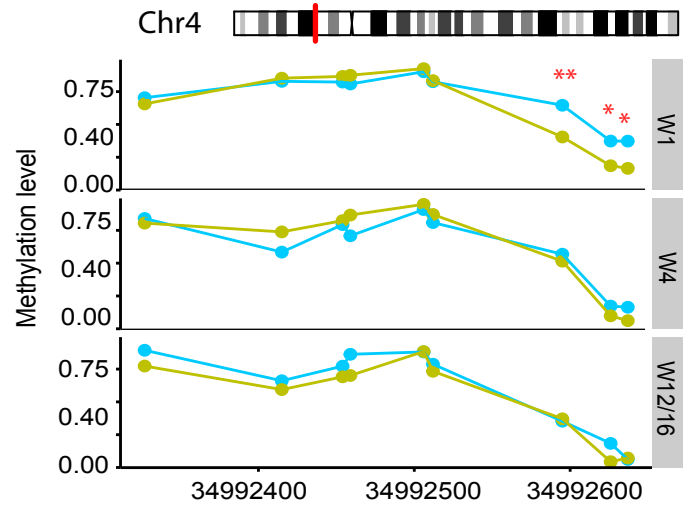

## Ube2a

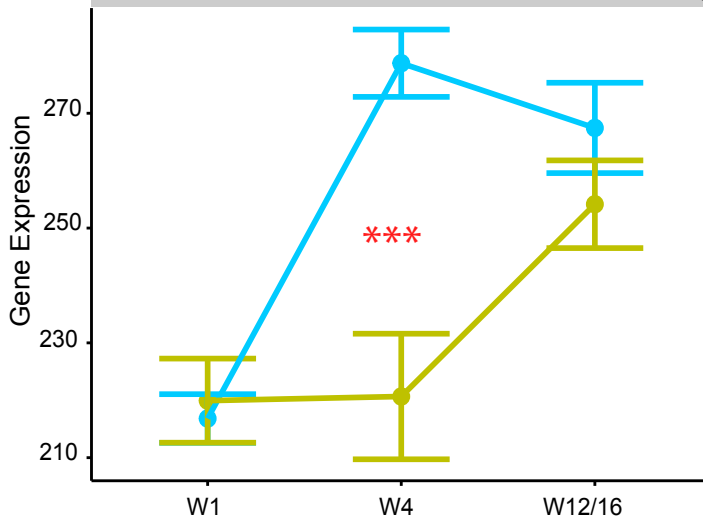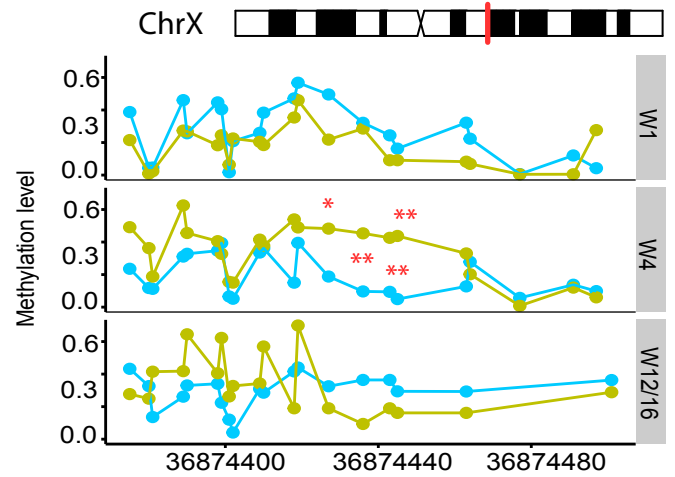

Supplement: Supplementary file 18 — Gene expression and DNA methylation levels of Mob3b (MOB kinase activator 3B) and Ube2a (Ubiquitin conjugating enzyme E2 A) genes and genomic loci in CONV-R and GF mice during postnatal development. (PDF 438 kb) [file 13073_2018_534_MOESM18_ESM.pdf]
